# Critical function of the necroptosis adaptor RIPK3 in protecting from intestinal tumorigenesis

**DOI:** 10.18632/oncotarget.10135

**Published:** 2016-06-17

**Authors:** Dominique Bozec, Alina C. Iuga, Giulia Roda, Stephanie Dahan, Garabet Yeretssian

**Affiliations:** ^1^ Immunology Institute, Icahn School of Medicine at Mount Sinai, New York, NY 10029, USA; ^2^ Division of Clinical Immunology, Department of Medicine, Icahn School of Medicine at Mount Sinai, New York, NY 10029, USA; ^3^ Department of Pathology and Cell Biology, Columbia University Medical Center, New York, NY 10032, USA; ^4^ The Leona M. Harry B. Helmsley Inflammatory Bowel Disease Center, The Henry D. Janowitz Division of Gastroenterology, Icahn School of Medicine at Mount Sinai, New York, NY 10029, USA; ^5^ Gastroenterology Unit, S. Orsola-Malpighi Hospital, Bologna, Italy; ^6^ Sobi, Inc., Waltham, MA 02452, USA; ^7^ Tisch Cancer Institute, Icahn School of Medicine at Mount Sinai, New York, NY 10029, USA

**Keywords:** colorectal cancer, IBD-related CRC, necroptosis, RIPK3

## Abstract

Necroptosis is a programmed form of non-apoptotic cell death that requires the kinase activity of the receptor interacting protein kinase 3 (RIPK3). Although *in vitro* data suggests that cancer cells lacking expression of RIPK3 are invasive, the physiological role of RIPK3 in a disease-relevant setting remains unknown. Here we provide evidence that RIPK3 has a critical role in suppressing colorectal cancer (CRC). RIPK3-deficient mice were highly susceptible to colitis-associated CRC and exhibited greater production of pro-inflammatory mediators and tumor promoting factors. Tumorigenesis in RIPK3-deficiency resulted from uncontrolled activation of NF-κB, STAT3, AKT and Wnt-β-catenin signaling pathways that enhanced the ability of intestinal epithelial cells (IECs) to aberrantly proliferate in the face of the sustained inflammatory microenvironment and promote CRC. We found that RIPK3 expression is reduced in tumors from patients with inflammatory bowel diseases, and further confirmed that expression of RIPK3 is downregulated in human CRC and correlated with cancer progression. Thus, our results reveal that the necroptosis adaptor RIPK3 has key anti-inflammatory and anti-tumoral functions in the intestine, and define RIPK3 as a novel colon tumor suppressor.

## INTRODUCTION

Uncontrolled intestinal inflammation, such as the one observed during inflammatory bowel disease (IBD), is a major risk factor for colorectal cancer (CRC) development [1, 2]. CRC is a frequent malignant tumor and the second leading cause of cancer-related death in developed countries [3]. Given the complex organization of the intestinal epithelium, cell death and renewal need to be tightly regulated as inappropriate cell death responses lead persistently to the development of intestinal inflammation and tumorigenesis [4, 5]. Increasing evidence underscores that excessive inflammatory conditions in the gut can initiate genetic alterations leading to neoplastic transformation of colonic epithelial cells, aberrant proliferation, angiogenesis and invasiveness [2, 6]. Thus, it is important to uncover the molecular mechanisms of inflammation-mediated CRC formation and progression, and to understand how specific molecular features of tumors dictate aberrant inflammatory responses.

Programmed necrosis or “necroptosis” is a non-apoptotic form of cell death that requires the kinase activity of the central adaptor receptor interacting protein kinase 3 (RIPK3) and has similar features of necrosis [7, 8]. Necroptosis has emerged as an important regulator of host immunity to pathogens and inflammation. Various endogenous and exogenous stimuli can trigger necroptosis through engagement of the tumor necrosis factor (TNF) receptor-like death receptors, Toll-like receptors (TLRs) 3 and 4, interferon (IFN)-receptors, and DNA-dependent activator of IFN-regulatory factors (DAI) [9-11]. These pathways promote the interaction of RIPK3 and the upstream kinase RIPK1 via their respective RIP-homotypic interaction motifs (RHIMs) and the formation of an amyloid-like RIPK1/RIPK3 necrosome complex [12]. Activation of RIPK3 leads to the recruitment and phosphorylation of mixed-lineage kinase domain-like (MLKL), which in turn forms oligomers that translocate to the plasma membrane and impair membrane integrity [13, 14].

Necroptosis and apoptosis share several common molecular components, and have been suggested to play a critical role in the pathogenesis of several inflammatory diseases including IBD [15, 16]. However, apoptosis depends on caspase activation and necroptosis is negatively regulated by caspases. Recent studies have put forward a new kinase-independent function of RIPK1 in maintaining intestinal barrier integrity and homeostasis [17, 18]. RIPK1 in intestinal epithelial cells (IECs) has been shown to prevent intestinal inflammation by directly inhibiting apoptosis driven by activation of caspase-8 and death-receptor adaptor protein Fas-associated death domain (FADD), as well as blocking RIPK3-dependent necroptosis [17, 18]. RIPK1 also procures cell survival during intestinal injury and inflammation in response to innate immune signals and cytokines such as TNFα, IFNβ, IFNγ, and the TLR3 ligand Poly (I:C) [17-20]. Interestingly, it has been reported that aberrant necroptosis activation, mediated by RIPK1-kinase function or RIPK3, can provoke loss of barrier integrity and subsequent intestinal inflammation [21-23]. In fact, loss of caspase-8 or FADD specifically in the intestinal epithelium sensitizes IECs to necroptosis and chronic inflammatory disease that is rescued by concomitant deletion of RIPK3 [22, 23]. Furthermore, abnormal levels of RIPK3 have been found in the intestinal epithelium of both adult and pediatric Crohn's disease patients [22, 24]. Altogether, these data indicate that inappropriate activation of necroptosis and subsequent loss of barrier integrity may account for the chronic intestinal inflammation underlying the pathogenesis of IBD.

RIPK3 has been shown to play a necroptosis-independent function in intestinal inflammation [25]. It has been reported that RIPK3 within dendritic cells (DCs) promotes injury-induced inflammation and tissue repair in the dextran sulfate sodium (DSS) model of colitis, partly through an IL-23, IL-1β, and IL-22 axis. Lack of RIPK3 in these cells was found to impair nuclear translocation of NF-κB subunit RelB-p50 and caspase-1-mediated pro-IL-1β processing [25]. More recently, RIPK3 expression has been shown to be downregulated in human CRC tissues when compared to adjacent normal tissues [26]. These findings correlate with observations that most cancer cell lines commonly used in the laboratory do not express RIPK3, which in response to chemotherapeutics represses programmed necrosis [27]. Moreover, overexpression of RIPK3 has been reported to suppress proliferation, migration and invasion of CRC cell lines [26]. These results support the idea that RIPK3 is critical in CRC as its expression might improve response to chemotherapy, and limit colorectal tumor initiation and progression. Yet, the implication of RIPK3 in the pathogenesis of CRC in more physiological settings remains unclear and whether RIPK3-dependent necroptosis has tumor suppressive functions needs to be uncovered.

In this study, we explore the physiological function of the necroptosis adaptor RIPK3 in CRC. We report that the expression of RIPK3 is decreased in the colon of CRC patients and in tumors from patients with IBD. We demonstrate that *Ripk3^−/−^* mice were highly susceptible to colitis-associated CRC and showed greater production of pro-inflammatory and tumor promoting factors. The increase in tumorigenesis was linked to overt IEC proliferation in the colons of *Ripk3^−/−^* mice. Together, these findings uncover RIPK3 as a critical tumor suppressor during intestinal inflammation and colitis-associated CRC.

## RESULTS

### RIPK3 attenuates progression and development of inflammation-driven CRC

**Figure 1:**
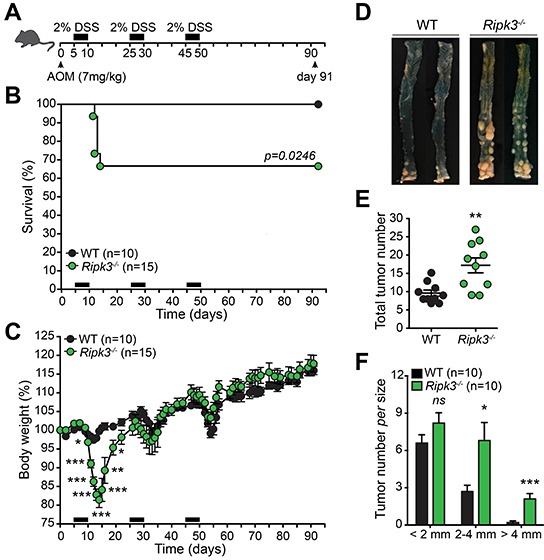
RIPK3 is critical for protecting against colitis-associated CRC **A.** Schematic representation of the AOM-DSS treatment. **B.** Kaplan-Meier survival curve of wild-type (WT; n=10) and *Ripk3^−/−^* (n=15) mice throughout the AOM-DSS regimen based on severe body weight loss as a consequence of excessive intestinal inflammation. *P* value was determined with Log-rank Mantel-Cox test. **C.** Body weight loss of WT and *Ripk3^−/−^* mice treated as in (B). **D.** Photographs of gross representative appearance of the colons of mice from both genotypes on day 91 post-treatment with AOM-DSS. Total tumor number **E.** and size **F.** in WT and *Ripk3^−/−^* mice. n, number of mice. *p<0.05, **p<0.01, ***p<0.001; *ns*, not statistical significance (two-tailed Student's t-test). Data represent mean ± SEM of two independent experiments.

*Ripk3^−/−^* mice have been recently shown to be highly susceptible to DSS-induced colitis [25]. It has been further described that RIPK3 in DCs controls pro-inflammatory cytokine production by promoting NF-κB activation and engaging caspase-1-mediated IL-1β release [25]. These observations prompted us to evaluate the role of RIPK3 in the initiation and progression of inflammation-driven CRC. To do so, Wild-type (WT) and *Ripk3^−/−^* mice were subjected to a single injection of the carcinogen azoxymethane (AOM) followed by three cycles of 2% DSS (Figure 1A), an established model of inflammation-driven CRC [28]. Notably, *Ripk3^−/−^* mice were highly susceptible to colitis when compared to WT mice, as 35% of these mice died and exhibited dramatic body weight loss after only one cycle of DSS treatment (Figure 1B and 1C). The mice that survived the first cycle of DSS-injury recovered and no further differences in body weight were observed between WT and *Ripk3^−/−^* mice throughout the remaining treatment period (Figure 1C), probably due to enhanced proliferation of IECs with pro-tumorigenic capabilities in the absence of RIPK3. Thirteen weeks after AOM-DSS regimen (day 91), lack of RIPK3 promoted marked tumor growth in the middle and distal part of the colon (Figure 1D and 1E), which closely mirrors the pattern seen in human CRC. Furthermore, *Ripk3^−/−^* mice developed higher number of larger tumors (Figure 1F). Collectively, these data indicate that RIPK3-deficiency contributes to increased tumor development and progression in the colon and imply that RIPK3 has an important protective function during colitis-associated CRC.

### Absence of RIPK3 enhances the development of more advanced colonic dysplasia

**Figure 2:**
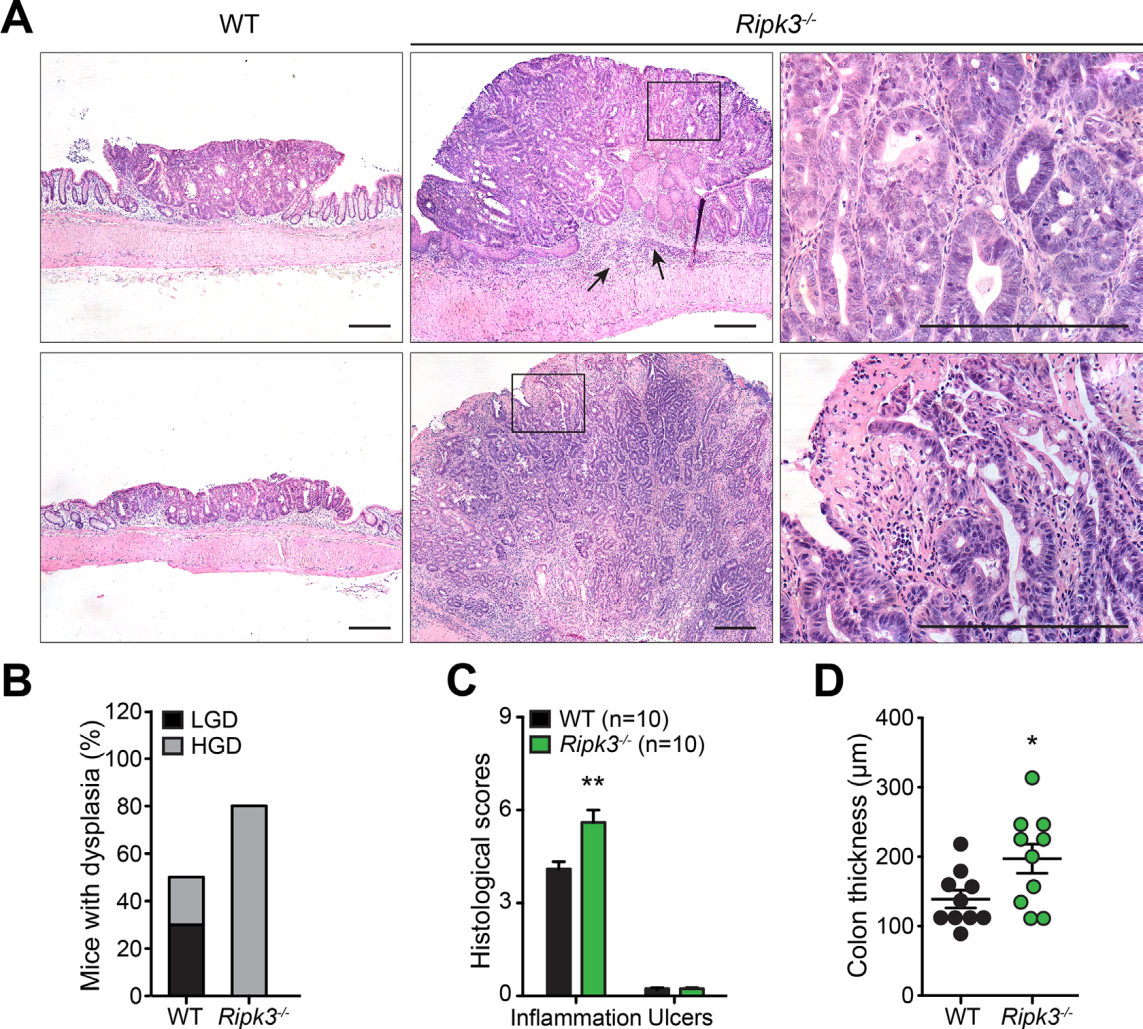
RIPK3-deficient mice are prone to DSS-induced colon tumor development **A.** Hematoxylin and eosin (H&E, original magnification ×4) staining of colon tissue section from WT and *Ripk3^−/−^* mice treated with AOM-DSS. Scale bars, 250 μm. Black arrows denote inflammation. Magnified views of the marked black areas are shown on the right panels. Scale bars, 250 μm. **B.** Percentage of mice with dysplasia. **C.** Pathology scores evaluating colon tissue inflammation and ulceration of the indicated mice on day 91 post-AOM-DSS. **D.** Colon thickness measured in uninvolved non-tumor areas using H&E stained tissue sections. n, number of mice. *p<0.05, **p<0.01 (two-tailed Student's t-test). Data represent mean ± SEM from two independent experiments.

In IBD-related CRC, cancer evolves through a neoplastic process that requires the development of dysplastic changes in the flat mucosa or in polypoid elevated lesions [29]. Likewise, combined treatment of mice with AOM and DSS generates neoplasms in the colonic mucosa via induction of dysplastic lesions [30]. To define whether lack of RIPK3 affects the morphological basis of tumor occurrence, Hematoxylin and eosin (H&E) stained colon tissue sections from WT and *Ripk3^−/−^* mice treated with AOM-DSS were assessed for histopathological features of CRC. While WT colorectal adenomas showed mainly low-grade of dysplasia depicted by classic tubular structures and moderately distorted glands, *Ripk3^−/−^* colorectal neoplasms displayed markedly abnormal glandular proliferation with cribriform architecture indicative of a higher level of dysplasia and progression to well-differentiated intra-mucosal adenocarcinomas (Figure 2A and 2B). No evidence of distal metastasis in the spleen, lungs, liver, or bone marrow was noted in mice sacrificed at day 91 of the AOM-DSS regimen. RIPK3-deficient animals with high-grade dysplasia and intra-mucosal adenocarcinomas had significantly more overall inflammation scores than WT animals (Figure 2C), which could also be appreciated by the increased colon thickness in the non-neoplastic colon (Figure 2D) and the enhanced tumor-underlying transmural inflammation seen in *Ripk3^−/−^* mice (Figure 2A; arrows). These observations further underscore that the pattern of neoplasms in *Ripk3^−/−^* mice is reminiscent of cancers arising in human IBD.

### Tumorigenesis in *Ripk3^−/−^* mice is mediated by excessive inflammation

**Figure 3:**
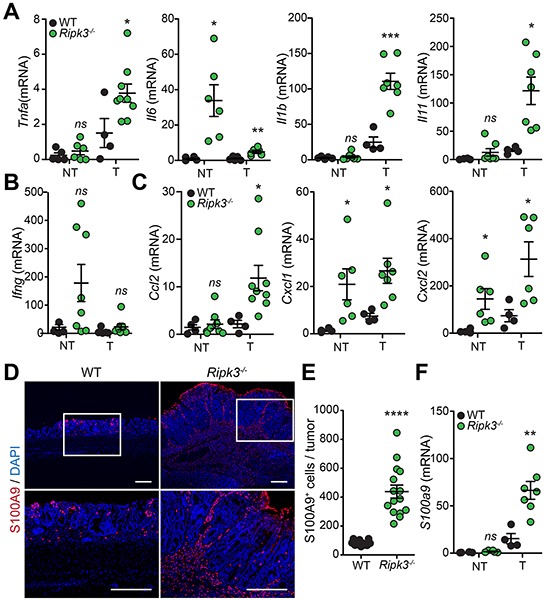
Loss of RIPK3 enhances colon inflammation and immune cell infiltration during tumor development Relative expression levels of indicated cytokine **A, B.** and chemokine **C.** mRNAs extracted from normal non-tumor (NT) and tumor (T) colon tissues from WT and *Ripk3^−/−^* mice treated with AOM-DSS. *p<0.05, ***p<0.001; *ns*, not statistical significance (two-tailed Student's t-test). Data represent mean ± SEM from two independent experiments; n ≥ 4. **D.** Representative immunofluorescence staining of S100A9 in colon tissue sections of WT and *Ripk3^−/−^* mice treated with AOM-DSS. Neutrophils stained with S100A9 (red) and nuclei with DAPI (blue). Magnified views of the marked white areas are shown in the lower panels. Scale bars, 250 μm. **E.** Quantification of S100A9^+^ cells in the colon tumors of WT and *Ripk3^−/−^*. Each data point denotes the average number of S100A9^+^ cells *per* tumor. ****p<0.0001 (two-tailed Student's t-test). Data represent mean ± SEM from three independent experiments. **F.** Real-time qPCR analysis of *S100a9* gene expression in NT and T tissues from the same mice as in (A). **p<0.01; *ns*, not statistical significance (two-tailed Student's t-test). Data represent mean ± SEM from two independent experiments; n ≥ 4.

RIPK3-deficient mice displayed markedly increased colon inflammation (Figure 2C and 2D) and adenomatous growth showing malignant transformation (Figure 2A and 2B). These types of tumors are frequently infiltrated with immune cells (e.g. myeloid cells and T lymphocytes) that prompt tumor-promoting inflammation [6]. We therefore postulated that RIPK3 might protect from colitis-driven CRC by dampening immune cell infiltration, activation and inflammatory responses. To establish whether inflammation is a potential mechanism contributing to the increased tumorigenesis in *Ripk3^−/−^* mice, we examined the expression of pro-inflammatory cytokines and chemokines in tumors and non-tumoral colonic tissues derived from WT and *Ripk3^−/−^* mice at day 91 after AOM-DSS, as these are key players in CRC. Real time qPCR analyses delineate increased transcription of genes encoding cytokines such as TNFα, IL-6, IL-1β, and IL-11 in colonic tumors of *Ripk3^−/−^* mice when compared to WT mice (Figure 3A), whereas the expression of IFNγ, which along its pro-inflammatory role has tumor-suppressing capabilities, remained unchanged in colonic tumors (Figure 3B). The levels of the chemokines CCL2, CXCL1 and CXCL2 were also significantly elevated in RIPK3-deficient tumors (Figure 3C). Interestingly, IL-6, CXCL1 and CXCL2 were also up-regulated in non-tumoral colon tissues derived from *Ripk3^−/−^* mice as an indication of increased inflammation and chemotaxis throughout the entire colon (Figure 3A and 3C).

Chronic inflammation contributes to tumor initiation in colitis-associated CRC, and infiltration of immune cells that play a crucial role during tumorigenesis. CCL2, CXCL1 and CXCL2 expressed by IECs, stromal and tumor cells during CRC possess pro-tumoral capacities since they chemoattract myeloid cells including neutrophils and tumor-associated macrophages, and stimulate angiogenesis [31, 32]. To unravel whether enhanced tumorigenesis in *Ripk3^−/−^* mice is associated with hyper-inflammatory responses, infiltration of S100A9 expressing inflammatory cells was assessed along the deep edges of colorectal tumors of WT and *Ripk3^−/−^*mice. S100A9 present in neutrophils and other myeloid cells has been reported to be expressed in human CRC and to coincide with tumor progression and invasion [33, 34]. In line with these observations, the number of S100A9^+^ neutrophils infiltrating into the colon tumors was significantly higher in *Ripk3^−/−^* mice relative to WT mice (Figure 3D and 3E). Furthermore, infiltration of neutrophils in RIPK3-deficient tumors correlated with enhanced mRNA expression of S100A9 (Figure 3F). These results highlight the significance of RIPK3 in dampening chronic inflammation that resides at the basis of CRC and suggest that infiltration of pro-inflammatory immune cells may provide signals that promote tumorigenesis in the absence of RIPK3.

### RIPK3 is a colon tumor suppressor

**Figure 4:**
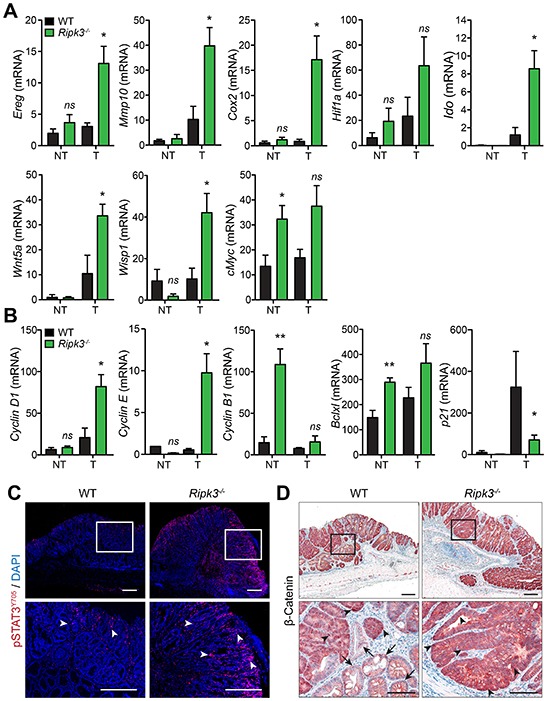
RIPK3 controls intestinal expression of angiogenic, mitogenic, and pro-tumorigenic genes **A, B.** Relative expression levels of angiogenic, mitogenic, and pro-tumorigenic genes in normal non-tumor (NT) and tumor (T) colon tissues from WT and *Ripk3^−/−^* mice treated with AOM-DSS. *p<0.05, **p<0.01; *ns*, not statistical significance (two-tailed Student's t-test). Data represent mean ± SEM from two independent experiments; n ≥ 4. **C.** Immunofluorescence staining of phosphorylated-STAT3 (pSTAT3^Y705^) in colon tumors of WT and *Ripk3^−/−^* mice treated with AOM-DSS. Cells stained with pSTAT3^Y705^ are depicted in red and by white arrowheads and nuclei stained with DAPI (blue). Magnified views of the marked white areas are shown in the lower panels. Scale bars, 250 μm. **D.** Immunohistochemical analysis of β-Catenin in WT and *Ripk3^−/−^* colon tumors. Black arrows denote membrane staining of β-Catenin and arrowheads indicate strong nuclear/cytosolic staining of β-Catenin, representative of activation of the Wnt signaling pathway following AOM-DSS. Magnified views of the marked black areas are shown in the lower panels. Scale bars, 250 μm and 100 μm.

Because *Ripk3^−/−^* mice exhibited higher tumor burdens accompanied with enhanced production of cytokines and chemokines (Figure 3), we next hypothesized that RIPK3 suppresses tumor development and progression by negatively regulating inflammatory mediators with pro-tumorigenic effects. In fact, growing evidence demonstrates that cytokines and chemokines drive the expression of pro-tumorigenic growth factors that modulate both neoplastic cells and the tumor microenvironment [6]. To characterize the nature of the deregulated tumorigenic signals in *Ripk3^−/−^* mice, we examined the expression of multiple angiogenic, mitogenic and tumorigenic factors in the intestine. Expression of genes involved in angiogenesis and mitogenic responsiveness, comprising the EGFR ligand Epiregulin (*Ereg*), matrix metalloproteinase 10 (*Mmp10*), and cyclooxygenase 2 (*Cox2*) were up-regulated in colon tumors of *Ripk3^−/−^* mice (Figure 4A). Importantly, the levels of key molecules associated with tumor promotion and progression were also increased in tumors of *Ripk3^−/−^* mice relative to WT mice, such as the genes encoding hypoxia-inducible factor 1 alpha (*Hif1a*), Indoleamine 2,3-dioxygenase (*Ido*), Wingless-type MMTV integration site family member 5A (*Wnt5a*), and Wnt1-inducible signaling pathway protein 1 (*Wisp1*) (Figure 4A). Moderate alterations were detected in the transcript levels of the *Hif1a* and *cMyc* (Figure 4A), however the expression of various genes involved in cell cycle, such as *Cyclin D1*, *Cyclin E*, and the cyclin-dependent kinase inhibitor *p21*, were markedly modulated in the RIPK3-deficient tumors (Figure 4B). No significant differences were detected between WT and *Ripk3^−/−^* mice in non-tumoral colon tissues, except for the transcript levels of *cMyc*, the anti-apoptotic *Bclxl* and *Cyclin B1* (Figure 4A and 4B). These findings are in agreement with the observed increase in dysplasia incidence and intra-mucosal adenocarcinoma formation in *Ripk3^−/−^* mice shown in Figures 1 and 2.

The expression of tumor promoting factors is often regulated by key transcription factors like STAT3 whose aberrant activation ultimately favors accumulation and promotion of neoplastic cells [35]. STAT3 has been implicated in intestinal inflammation and CRC, and its phosphorylation through gp130 receptor engagement by IL-6 and IL-11 enables its nuclear translocation and function [36, 37]. To examine whether RIPK3 modulates CRC through STAT3 signaling, phosphorylation of STAT3 on tyrosine residue 705 (STAT3^Y705^) was assessed in colonic tumors of WT and *Ripk3^−/−^* mice by immunofluorescence microscopy. Elevated levels of phosphorylated-STAT3 (pSTAT3) were evident in RIPK3-deficient *versus* WT mice (Figure 4C). In *Ripk3^−/−^* mice, pSTAT3 was strongly expressed in most epithelial cells, as well as in many stromal and tumor infiltrating cells, whereas in WT mice pSTAT3 was observed in few infiltrating cells (Figure 4C).

To further determine the high prevalence of tumor promoting factors in the absence of RIPK3, tumors from WT and *Ripk3^−/−^* mice were analyzed for the expression of β-catenin. Canonical Wnt-mediated β-catenin activation prompts target gene expression that regulates intestinal homeostasis and the generation of intestinal crypts [38, 39]. Moreover, functional mutations in the β-catenin gene play an essential role in the progression of human CRC as well as AOM-induced colonic tumors in mice [39-41]. Indeed, staining for β-catenin revealed a strong and uniform translocation of β-catenin to the cytoplasm and/or nucleus of IECs in the dysplastic areas of *Ripk3^−/−^*tumors (Figure 4D). In tumors of WT mice, β-catenin expression pattern was more heterogeneous as within the same group of neoplastic cells some retained its membrane expression (arrows) while other adjacent cells showed cytoplasmic and/or nuclear expression of β-catenin (arrowheads; Figure 4D). Together, these findings support our hypothesis that RIPK3 has anti-tumor properties and that lack of RIPK3 promotes the development of intestinal neoplasia through STAT3- and β-catenin-mediated transcription of angiogenic, mitogenic, tumorigenic and cell cycle regulators.

### RIPK3 controls overt proliferation of colon epithelial cells

**Figure 5:**
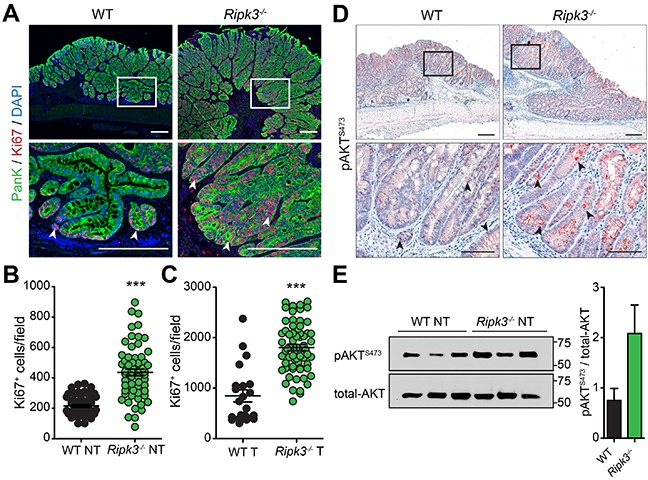
RIPK3 inhibits hyper-proliferation of colon epithelial cells **A.** Immunofluorescence staining of Ki67 in colon tissue sections of WT and *Ripk3^−/−^* mice treated with AOM-DSS. IECs stained with Pan-Keratin (PanK; green) and nuclei with DAPI (blue). Proliferative Ki67^+^ colon epithelial cells (red) are denoted by white arrowheads. Magnified views of the marked white areas are shown in the lower panels. Scale bars, 250 μm. Quantification of Ki67^+^ cells in uninvolved NT **B.** and tumor **C.** tissue of WT and *Ripk3^−/−^* post-AOM-DSS. Each data point represents the average number of Ki67^+^ cells per field. ***p<0.001 (two-tailed Student's t-test). Data represent mean ± SEM from three independent experiments. **D.** Immunohistochemical analysis of phosphorylated-AKT (pAKT^S473^) in colon tumor epithelial cells of WT and *Ripk3^−/−^* mice on day 91 after AOM-DSS. Magnified views of the marked black areas are shown in the lower panels. Arrowheads indicate pAKT^S473^ expressing IECs (red). Scale bars, 250 μm and 100 μm. **E.** Immunoblot analysis and densitometric quantification of pAKT^S473^ and total-AKT in non-tumor (NT) colon tissues. Each lane represents an individual mouse. Bar graphs denote quantification of each lane (n=3 mice per genotype). Data represent mean ± SEM from two independent experiments.

Many pro-tumorigenic cytokines expressed during colitis-associated CRC have proliferative and survival effects on tumor initiating IECs. Given the increased expression of pro-inflammatory and pro-tumorigenic factors in colonic tumors of *Ripk3^−/−^* mice (Figures 3 and 4), we wanted to examine whether RIPK3 regulates proliferation of IECs following AOM-DSS treatment. Staining with Ki67 specific antibodies did not reveal any significant differences in basal crypt proliferation rates between naïve WT and *Ripk3^−/−^* mice (data not shown). However, IEC proliferation within tumors of *Ripk3^−/−^* mice was significantly elevated relative to WT mice (Figure 5A-5C). Importantly, untransformed colon tissue and unaffected colon regions of *Ripk3^−/−^*mice also exhibited higher proliferation levels when compared to WT mice as measured by Ki67 staining (Figure 5B). These results demonstrate that RIPK3 suppresses proliferation of tumor promoting IECs by negatively regulating inflammatory and pro-tumorigenic signaling pathways.

To gain further insights into the mechanism underlying overt IEC proliferation in the absence of RIPK3, we analyzed the activity of AKT, a known regulator of epithelial homeostasis. The phosphatidylinositol-3-Kinase (PI3K)/AKT pathway is oncogenic; it transduces mitogenic signals by promoting β-catenin nuclear translocation and stability, and plays a central role in regulating IEC proliferation and differentiation [42, 43]. Consistent with the data that β-catenin is strongly translocated to the cytoplasm and/or nucleus of IECs in tumors of *Ripk3^−/−^* mice (Figure 4D), levels of phosphorylated-AKT on serine residue 473 (pAKT^S473^) dramatically increased in tumor IECs of *Ripk3^−/−^* mice but to a much lesser extent in WT mice (Figure 5D), reflecting a higher PI3K/AKT signaling. Similarly, greater AKT activity was observed in untransformed and non-tumoral mucosal lysates of *Ripk3^−/−^* mice compared with WT controls, as evidenced by immunoblotting for pAKT^S473^ and total-AKT (Figure 5E). Taken together, these findings indicate that the absence of RIPK3 creates a pro-tumorigenic microenvironment that influences unwarranted IEC proliferation and CRC progression, and further suggest that RIPK3 regulates IEC proliferation by suppressing AKT-β-catenin mediated signaling.

### RIPK3 in human IBD-associated CRC

**Figure 6:**
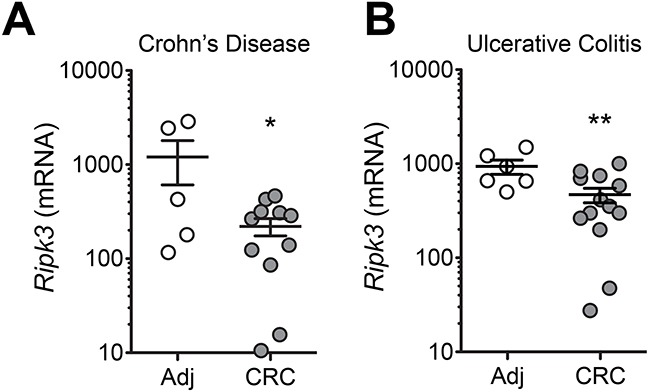
Decreased expression of *RIPK3* in colonic mucosa of patients with colitis-associated CRC Expression of *RIPK3* in colorectal cancer (CRC) or normal adjacent (Adj) tissue samples extracted from surgical specimens of Crohn's Disease **A.** and Ulcerative colitis **B.** patients. Data represent relative expression ± SEM. *p<0.05, **p<0.01 (two-tailed Student's t-test).

Patients with IBD, both ulcerative colitis (UC) and Crohn's colitis of Crohn's disease (CD), are at a high risk for the development of CRC depending on the duration of the disease and the extent of colorectal involvement [3]. Having displayed the involvement of RIPK3 in colitis-associated CRC in mice, we sought to determine whether the expression of RIPK3 was reduced in IBD-related CRC. IBD patients undergo a rigorous screening procedure to prevent CRC development, but little genomic and transcriptomic data are available using IBD-associated CRC and adjacent non-neoplastic tissues. It is important to mention that sporadic CRC and IBD-related CRC differ in the molecular features of tumorigenesis, however they both elicit an inflammatory response. To investigate the presence of RIPK3 in human IBD-associated CRC, a set of Crohn's disease- or ulcerative Colitis-associated CRC patient resections were examined in areas of tumor tissue and non-neoplastic adjacent tissue for RIPK3 expression by quantitative real-time PCR. Interestingly, *RIPK3* mRNA expression was significantly lower in tumor versus non-neoplastic tissue both in Crohn's disease and ulcerative colitis patient samples (Figure 6A and 6B). These observations highlight for the first time the involvement of RIPK3 in IBD-associated CRC development.

### Decreased *RIPK3* mRNA expression predicts poor prognosis of CRC patients

**Figure 7:**
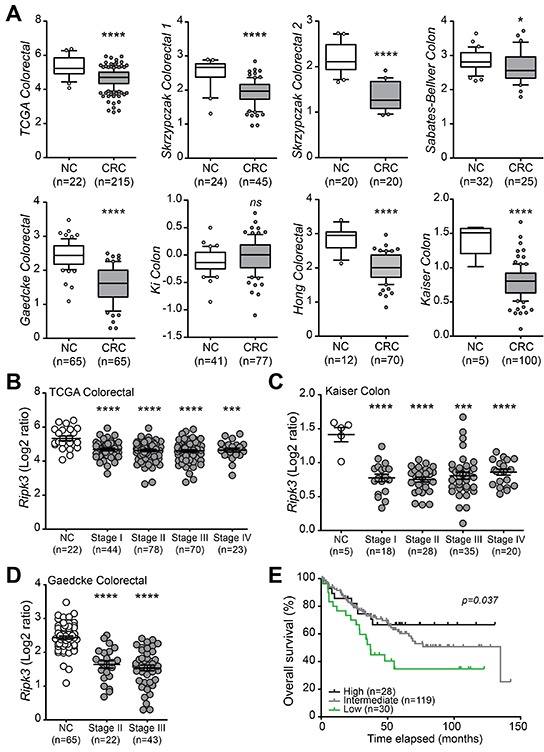
RIPK3 expression is downregulated in human CRC **A.** *RIPK3* mRNA expression in colorectal cancer (CRC) compared with normal colorectal (NC) tissues in eight gene expression microarray datasets accessed using the Oncomine database (https://www.oncomine.com/). Box-and-whiskers plots depict the distribution of *RIPK3* expression within each group presented as Log2 median-centered ratios and created using GraphPad Prism 5. The whiskers are drawn down to the 10^th^ percentile and up to the 90^th^. Points below and above the whiskers are drawn as individual dots. n, sample numbers. *p<0.05, ***p<0.001, ****p<0.0001; *ns*, not statistical significance (two-tailed Student's t-test). Assessment of *RIPK3* mRNA expression in different stages of CRC progression using the TCGA colorectal **B.**, Kaiser colon **C.** and Gaedcke colorectal **D.** databases. ***p<0.001, ****p<0.0001 (two-tailed Student's t-test). **E.** Kaplan-Meier analyses of *RIPK3* mRNA expression in CRC patients using the Smith Colorectal dataset from Oncomine. Overall survival curves representing high (top 15%), intermediate, and low (bottom 15%) gene expression of *RIPK3* based on Log2 median-centered ratio values. *P* value was determined with Log-rank Mantel-Cox test.

To determine gene expression levels of the necroptosis adaptor RIPK3 in CRC, differential expression of *RIPK3* mRNA was further analyzed in CRC and normal colorectal (NC) tissues in eight gene expression microarray datasets using the Oncomine® database. *RIPK3* transcript was significantly reduced in CRC when compared with NC tissues in seven out of eight datasets including TCGA Colorectal (NC/CRC=22/215); Skrzypczak Colorectal (24/45); Skrzypczak Colorectal 2 (20/20); Sabates-Bellver Colon (32/25); Gaedcke Colorectal (65/65); Hong Colorectal (12/70); and Kaiser Colon (5/100) (Figure 7A). No differences in *RIPK3* levels between CRC and NC tissues were found in the Ki Colon (41/77) dataset (Figure 7A; and Supplementary Table S1). Interestingly, in TCGA Colorectal and Kaiser Colorectal datasets, *RIPK3* was decreased in adenocarcinomas from different anatomical locations involving the cecum, colon, and rectum (Supplementary Table S1).

To further investigate the correlation between *RIPK3* gene expression and disease severity, TCGA Colorectal, Kaiser Colon and Gaedcke Colorectal datasets were examined for the expression levels of RIPK3. Results of these queries revealed that expression of *RIPK3* is decreased in all stages of CRC (stages I to IV) relative to healthy controls, and that the decline in RIPK3 levels correlates with increased clinical stage or the presence of metastases according to the tumor node metastasis (TNM) staging system (Figure 7B-7D).

To define whether reduced expression of RIPK3 has any prognosis significance in CRC, we employed the Smith Colorectal dataset in the Oncomine® platform that contains 177 CRC cases (Completed/Censored=73/104) and analyzed the survival of CRC patients with different *RIPK3* mRNA level. Interestingly, low expression of *RIPK3* was strongly linked with overall survival rate (*p* = 0.037), and subsequently with poor prognosis of CRC patients (Figure 7E), which is in agreement with recent observations describing association of low RIPK3 expression with TNM clinical stages in a cohort of CRC patients [26]. Altogether, these results indicate that downregulation of RIPK3 is a common feature of poor prognosis in CRC patients, and further suggest that RIPK3 has a suppressive function during CRC development and progression.

## DISCUSSION

Chronic inflammation seen in IBD patients is a predisposing factor of the onset and progression of CRC [3]. Initial studies have reported that abnormal activation of necroptosis contributes to human IBD [22, 24]. Necroptosis occurrence has also been associated with loss of barrier integrity and intestinal inflammation in mice [21-23]. Yet, the role of necroptosis in intestinal pathogenesis remains controversial and a growing body of evidence suggests that necroptosis is protective during intestinal inflammation [16-18]. A recent study proposed that RIPK3 is essential for injury-induced inflammation and tissue repair in the intestine [25]. Accordingly, another report found that RIPK3 is downregulated in human CRC tissues relative to adjacent normal tissues and that *in vitro* overexpression of RIPK3 blocks proliferation and invasion of CRC cell lines [26]. Despite these recent advances, the role of RIPK3 in regulating CRC initiation and progression in more physiological settings is still unknown. To the best of our knowledge, our results indicate for the first time that RIPK3 has tumor suppressor functions during inflammation-mediated CRC.

The expression of RIPK3 has been previously examined in human CRC and revealed that reduced RIPK3 levels correlated with poor outcome for CRC patients [26, 44]. Consistently, we found decreased expression of RIPK3 in human CRC following broad examination of CRC and normal colorectal tissues in the Oncomine® database. Seven out of eight public datasets revealed that RIPK3 levels were declined in human CRC when compared to healthy controls. These data also agree with earlier observations that RIPK3 expression is often silenced in cancer cells altering their response to chemotherapeutic agent-induced cell death [27, 44]. While one report proposed that RIPK3 expression is suppressed in cancer cells by promoter methylation [27], it is more likely that in solid tumors RIPK3 expression is inhibited by hypoxia, which has been implicated in promoting cell survival and angiogenesis during tumor progression [44]. Interestingly, the downregulation of RIPK3 in human CRC tissues predicted poor outcome of CRC patients, as revealed by the Kaplan-Meier survival analysis. RIPK3 also displayed a lower level of expression in all clinical stages of CRC (stages I to IV) relative to healthy controls, implying a potential role for RIPK3 in tumor development.

With the incidence of both Crohn's disease and ulcerative colitis being on the rise, the development of IBD-associated CRC is an increasingly important health concern [3]. Similar to CRC patients, we found that *RIPK3* mRNA was highly reduced in IBD-associated tumor tissues relative to adjacent non-tumoral tissues. These data collectively point towards a tumor suppressor function of RIPK3 in the onset and progression of IBD-associated CRC.

RIPK3 has been shown to be protective during intestinal inflammation induced by treatment of WT and *Ripk3^−/−^* mice with DSS [25]. Our data demonstrate that in the absence of RIPK3, colonic inflammation was increased as was the tumor incidence and size following AOM-DSS treatment, indicating that RIPK3 has a fundamental role in inhibiting intestinal inflammation and colitis-associated CRC development. RIPK3-deficient mice developed more and larger tumors than WT mice. These tumors displayed dysplastic lesions that progress from low- to high-grade dysplasia, and in some *Ripk3^−/−^* mice give rise to intra-mucosal adenocarcinomas. Also, *Ripk3^−/−^* animals with dysplasia had higher inflammation scores than WT mice with dysplasia, which is in line with the concept that inflammation-related genes, transcribed by one of the key transcription factors NF-κB, prominent in inflamed mucosa remain elevated in colonic neoplasms [6].

Throughout the multistep process of tumorigenesis in colitis-associated CRC, inflammatory cytokines drive activation of key pro-tumorigenic transcription factors (e.g. NF-κB and STAT3) in IECs to promote tumor cell proliferation and resistance to apoptosis [2]. NF-κB is activated downstream pattern-recognition receptors or by cytokines like TNFα and IL-1β. It has been shown that NF-κB is critical for the transition from inflammation to CRC malignancy through transcription of genes involved in survival, cell cycle progression, and inflammation [45]. Notably, persistent activation of NF-κB in IECs, thru expression of constitutively active IκB kinase β (IKKβ), has been associated with accelerated loss of heterozygocity by enhanced DNA damage and CRC development [46]. Concordantly, in a mouse model of AOM-DSS, deletion of IKKβ in IECs decreased tumor incidence without affecting tumor size or reducing inflammation, whereas deletion of IKKβ in myeloid cells strongly impaired inflammation and inhibited proliferation of pre-tumorigenic IECs [47]. RIPK3-deficient mice exhibited high activation of NF-κB in dysplastic and non-dysplastic areas of the colon evidenced by dramatic increase in the expression of IL-6, an iconic cytokine induced by NF-κB and responsible for CRC progression through IL-6-induced STAT3 signaling [35, 37]. Indeed, we found increased activation and phosphorylation of STAT3 in tumors of *Ripk3^−/−^* mice subjected to AOM-DSS when compared to WT mice. Similarly, expression level of IL-11, a member of the IL-6 family of cytokines, was increased in tumor tissues of *Ripk3^−/−^* mice, further emphasizing the effector functions of the IL-6-STAT3 and IL-11-STAT3 axes in our model. IL-11 has been previously described to be highly expressed in human CRC and to trigger tumorigenesis via oncogenic STAT3 activation [48].

Another evidence that linked NF-κB activation in the absence of RIPK3 with CRC progression was provided by our results showing dramatic increase in the expression of cytokines (TNFα and IL-1β) and chemokines (CCL2, CXCL1 and CXCL2). TNFα, produced by diverse cell types in the lamina propria, is a key mediator of inflammation and immunity. It is noteworthy that although TNFα has been described to be tumor suppressive, chronic expression of TNFα confers tumor promoting functions in the colon by acting on IECs to activate NF-κB-dependent tumorigenic cascades [49]. IL-6 can directly induce TNFα transcription and sustain chronic inflammation, yet it should be noted that cross-regulation between these cytokines is possible as TNFα can also directly induce IL-6 production through recruitment of immune cells and activation of NF-κB- and AP-1-dependent mechanisms. These observations are consistent with our data showing high levels of TNFα and greater tumor incidence in the colons of *Ripk3^−/−^* mice. We also found an enhanced recruitment of inflammatory cells, including S100A9^+^ neutrophils, to the colons of *Ripk3^−/−^* mice after AOM-DSS, which is in line with the role of these cells in up-regulating the Wnt-β-catenin pathway in tumor cells and CRC progression [34, 50]. Neutrophil infiltration during colitis-associated CRC is well appreciated to drive IL-1β release in the tumor microenvironment, another strong activator of NF-κB in IECs and tumor cells [51]. Nonetheless, IL-1β can also activate the Wnt-β-catenin signaling in CRC by AKT-dependent glycogen synthase kinase 3β (GSK3β) inactivation [52]. Remarkably, RIPK3-deficient mice exhibited a strong and uniform translocation of β-catenin to the cytoplasm and/or nucleus of IECs in the dysplastic areas of the colon. Based on our results, both TNFα and IL-1β emerge as important NF-κB-inducing cytokines and potent drivers of Wnt-β-catenin signaling in CRC when RIPK3 is not present.

A growing body of evidence further suggests that chronic activation of STAT3 and Wnt-β-catenin pathways induces expression of genes that promote IEC turnover and proliferation, DNA damage and CRC. Nevertheless, we do not rule out the involvement of other signaling pathways that also promote CRC development. In *Ripk3^−/−^* mice treated with AOM-DSS, we observed enhanced expression of several known STAT3-dependent genes, including *Mmp10*, *Cox2*, *Hif1a*, *Bclxl* and *cyclin D1*, that shape the tumor microenvironment and that contribute to CRC development by modulating apoptosis, angiogenesis and invasiveness of pro-tumorigenic IECs [36, 37]. Interestingly, RIPK3-deficient mice had increased Wnt-β-catenin signaling shown by induction of genes important for IEC survival, proliferation and invasiveness (*cMyc*, *Cox2* and *Wisp1*), and cell cycle progression (*Cyclin B1*, *Cyclin D1*, *Cyclin E* and *p21*) during colitis-associated CRC. The aberrant induction and translocation of β-catenin in the tumors of *Ripk3^−/−^* mice indicated that RIPK3-deficiency renders IECs highly proliferative, which correlated with the idea that constitutive activation of Wnt-β-catenin induces de-differentiation of IECs and facilitates tumorigenesis [53]. In accord, recent *in vitro* studies have revealed that overexpression of RIPK3 in CRC cell lines restrains cell proliferation and invasion [26]. Besides, we found that dysregulated proliferation of IECs in non-dysplastic and dysplastic areas of the colon in the absence of RIPK3 was accompanied with heightened activation and phosphorylation of AKT, further confirming the mechanism that RIPK3 protects against colitis-associated CRC by repressing excessive IEC proliferation.

Collectively, accelerated tumorigenesis in RIPK3-deficiency likely results from a combination of enforced sustained inflammation associated with persistent tissue damage, genome instability, and augmented ability of IECs to evade apoptosis and aberrantly proliferate in the face of the pro-inflammatory microenvironment. Our study shows that RIPK3 exerts tumor suppressor functions by negatively regulating activation of NF-κB, STAT3 and AKT, thereby protecting from aberrant IEC proliferation and CRC development. This study also provides first evidence using a physiologically relevant *in vivo* model of CRC and raises the possibility that RIPK3-mediated responses might act to retard tumor growth in the intestine and that necroptosis might be tumor-suppressive in the intestine. It is most likely that more work is required to clearly define whether RIPK3 directly communicates with the abovementioned signaling molecules, whether necroptosis-dependent or independent machinery is involved in CRC tumorigenesis, and whether increasing the expression of RIPK3 in the colon may be considered as a therapeutic strategy for human CRC and IBD-related CRC patients.

## MATERIALS AND METHODS

### Oncomine database analysis

Gene expression data of *RIPK3* was obtained from eight different CRC datasets using the Oncomine database (https://www.oncomine.com/) and standard procedures as previously described [54]. Fold change of *RIPK3* mRNA expression obtained from each CRC dataset was compared between CRC and normal colorectal (NC) tissues using the following filters: threshold fold change ≥ 1.5X; *p* value ≥ 1E-04; and gene rank in the top 10%. Expression values of *RIPK3* were sorted based on *p* values and presented in Log2 median-centered intensity ratios for eight CRC datasets including: TCGA Colorectal [55]; Skrzypczak Colorectal [56]; Skrzypczak Colorectal 2 [56]; Sabates-Bellver Colon [57]; Gaedcke Colorectal [58]; Ki Colon [59]; Hong Colorectal [60]; and Kaiser Colon [61]. Skrzypczak Colorectal 2 contains a subset of selected samples from Skrzypczak Colorectal further treated with micro-dissection. Expression of RIPK3 in patients stratified according to tumor staging defined by the American Joint Committee on Cancer TNM staging system was based on TCGA Colorectal, Kaiser Colon and Gaedcke Colorectal gene expression profiling datasets. For survival-associated expression analysis of RIPK3, Smith Colorectal dataset in the Oncomine platform was employed [62]; female and male patients were classified as high (top 15%), intermediate and low (bottom 15%) relative to the group median of *RIPK3* mRNA expression.

### Patient tissues

Disposed anonymous surgical specimens from patients with IBD undergoing bowel resection for CRC at the Mount Sinai Medical Center were used. Messenger RNA was extracted from tumors (CRC) or normal mucosal tissue adjacent to the tumor (Adj, >10 cm from the tumor) derived from patients with either Crohn's Disease (CD) and Ulcerative Colitis (UC).

### Mice

Wild-type (C57BL/6J) mice were purchased from Jackson Laboratories and maintained at the Icahn School of Medicine at Mount Sinai for more than 10 generations. *Ripk3^−/−^* mice on C57BL/6J background were a gift from V. Dixit (Genentech; [63]). Mice were used at 7-9 weeks of age. Experiments were carried out using age and gender matched groups. Animals were housed under specific-pathogen free (SPF) conditions. The Institutional Animal Care and Use Committee (IACUC) of the Icahn School of Medicine at Mount Sinai approved all the procedures performed in this study.

### Induction of CRC

For the induction of colitis-associated CRC, wild-type (WT) and *Ripk3^−/−^* mice were injected intraperitoneally with azoxymethane (AOM, Sigma-Aldrich) at 7 mg/kg body weight. At day 5 after AOM injection, mice were given three cycles of 2.5% dextran sulfate sodium (DSS; MW 36,000-50,000 from MP Biomedicals) in drinking water for 5 days followed by regular drinking water for 14 days [28]. Mice were monitored daily for body weight loss to assess disease progression, and mice that lost more than 20% of their initial body weight were considered dead and sacrificed. Following completion of the AOM-DSS regimen (day 91), mice were sacrificed; colons were removed from animals, flushed with cold PBS and cut longitudinally. A picture of the colon was taken with a Coolpix L28 camera (Nikon). Number and size of tumors present per mouse were blindly counted as previously described [28, 64].

### Histopathology, immunohistochemistry and immunofluorescence

Colon tissues were fixed overnight in 10% phosphate-buffered formalin, embedded in paraffin and cut in 4 μm sections. Hematoxylin & eosin (H&E) stained sections were scored blindly for the amount of inflammation and ulcerations [65]. Grades of colonic mucosal dysplasia, low (LGD) or high (HGD) grade dysplasia, were defined according to previously described criteria [66]. Colon thickness was estimated in uninvolved non-tumor areas throughout the entire colon of WT and *Ripk3^−/−^* mice, and values corresponding to the average thickness in μm per mouse are represented. Formalin-fixed paraffin-embedded tissue sections were de-waxed and rehydrated, incubated in Dako antigen retrieval solution (DAKO) and boiled for 20 min in a pressure cooker. For β-catenin, slides were deparaffinized using Tris-EDTA and boiled for 50 min in a pressure cooker. For immunohistochemistry (IHC), endogenous peroxidase activity was quenched with 1.5% hydrogen peroxide in methanol for 15 min at room temperature. Staining was performed according to the manufacturer's protocol using a rabbit Histostain-Plus kit (Life Technologies) and incubation times with 3-amino-9-ethylcarbazole (AEC) substrate were equal for all samples. Tissues were incubated overnight with primary antibodies against phosphorylated-AKT (pAKT^S473^; Cell Signaling Technologies) or β-catenin (Cell Signaling Technologies) in phosphate buffer saline (PBS) containing 0.1% Triton X-100. Slides were counterstained with Mayer's Hematoxylin Solution (Sigma-Aldrich) and then mounted with a coverslip. For immunofluorescence, deparaffinized sections were blocked in 10% BSA, Tris-buffered saline, and 0.3% Triton X-100 for 30 min at room temperature. Cell proliferation was evaluated by incubating the tissues with primary antibodies against Ki67 and pan-cytokeratin (Abcam) for 1 hr at room temperature and followed by 1 hr incubation with Alexa-488 coupled anti-mouse IgG1 and Alexa-594 conjugated anti-rabbit antibodies (Life Technologies), respectively. STAT3 activation was assessed on tissue sections incubated overnight at 4°C with a primary antibody against phosphorylated-STAT3 (pSTAT3^Y705^; Cell Signaling technologies) followed by 1 hr incubation with Alexa-594 conjugated anti-rabbit antibody (Life Technologies). Similarly, expression of S100A9 was evaluated by probing the tissues with primary antibody against S100A9 (R&D Systems) overnight at 4°C followed by 1 hr incubation with Alexa-594 conjugated anti-goat IgG antibody (Life Technologies). All tissues were incubated with 4′, 6′-diamidino-2-phenylindole (DAPI) for 10 min for DNA staining, washed and mounted with a coverslip using Fluoromount-G (Southern Biotech). Slides were analyzed in a blinded fashion for S100A9^+^ cells by assessing the number of cells per tumor, and for Ki67^+^ cells by counting cells in different fields covering the entire colon section using the ImageJ/FIJI software. All slides were examined using a Nikon Eclipse Ni series microscope and images captured at original magnification x4, and x10.

### Quantitative real-time RT-PCR

Total RNA was extracted from normal non-tumor (NT) and tumor (T) colon tissues with Trizol reagent (Life Technologies), followed by isopropanol precipitation. We reverse-transcribed 2 mg total RNA to complementary DNA (cDNA) by using M-MLV reverse transcriptase and random hexamers (Life technologies) according to the manufacturer's protocol. qRT-PCR with reverse transcription was performed using Absolute Blue qPCR SYBR Green mix (Thermo Scientific) and on a VIIA7 real-time PCR system (Applied Biosystems; Life technologies). PCR results were normalized to *L32* expression and relative expressions calculated using the ΔΔCT method. Primer sequences used in this study are summarized in Supplementary Table S2.

### Immunoblotting

SDS-polyacrylamide gels and immunoblotting (IB) were performed in accordance with previously described standard protocols [67]. For activation of AKT in colonic tissues, 1-2 cm uninvolved normal non-tumor (NT) tissue was collected from the distal colon of each mouse, washed twice with PBS and placed in NP-40 buffer (20 mM Tris-HCl (pH 8.0), 150 mM KCl, 10% glycerol, 5 mM MgCl_2_, and 0.1% NP-40) supplemented with protease and phosphatase inhibitor tablets (Roche). Tissue samples were homogenized, and total protein concentration was evaluated in the lysates using the Bradford protein assay (Bio-Rad). Antibodies against phosphorylated-AKT (pAKT^S473^) and total-AKT were purchase from Cell Signaling Technologies, and secondary antibodies were from Jackson ImmunoResearch Laboratories.

### Statistical analysis

All results are presented as mean ± SEM. Prism5 (GraphPad) software was used for all statistical tests. Statistical significance was determined by two-tailed Student's *t-test* for comparison of two groups, and Log-rank Mantel–Cox test for survival data. Differences were considered statistically significant when *P* ≤ 0.05. *P* values are indicated by *P < 0.05, **P < 0.01, ***P < 0.001 and ****P < 0.0001.

## SUPPLEMENTARY TABLES


