# Supplementary material for: Critical function of the necroptosis adaptor RIPK3 in protecting from intestinal tumorigenesis
Source: Oncotarget. 2016 Jun 17;7(29):46384–400. doi: 10.18632/oncotarget.10135 (PMC5216805; doi:10.18632/oncotarget.10135)
Supplement: Supplementary file 1 [file oncotarget-07-46384-s001.pdf]

# Critical function of the necroptosis adaptor RIPK3 in protecting from intestinal tumorigenesis

## SUPPLEMENTARY TABLES

**Supplementary Table S1: Statistical analysis of RIPK3 expression in CRC in eight different microarray databases and from various anatomical localizations**

| | Cancer *vs.* Normal | |
|---|---|---|
| **Database/cancer type** | **Fold change** | **p value** |
| **Skrzypczak CRC1** | | |
| - Colorectal Adenocarcinoma | -1.502 | 1.3E-07 |
| **Skrzypczak CRC2** | | |
| - Colorectal Adenocarcinoma | -1.791 | 5.1E-10 |
| **Sabates Belver colon** | | |
| - Colon Adenocarcinoma | -1.142 | 4.7E-02 |
| - Rectum Adenocarcinoma | -1.264 | 2.5E-02 |
| **Ki Colon** | | |
| - Colon Adenocarcinoma | 1.076 | 7.4E-02 |
| **Gaedcke CRC** | | |
| - Rectum Adenocarcinoma | -1.816 | 9.1E-19 |
| **Hong CRC** | | |
| - Colorectal Adenocarcinoma | -1.793 | 4.8E-07 |
| **Kaiser CRC** | | |
| - Cecum Adenocarcinoma | -1.495 | 8.6E-04 |
| - Colon Adenocarcinoma | -1.539 | 1.4E-03 |
| - Rectum Adenocarcinoma | -1.558 | 7.9E-04 |
| **TCGA CRC** | | |
| - Cecum Adenocarcinoma | -1.695 | 7.2E-05 |
| - Colon Adenocarcinoma | -1.636 | 1.5E-05 |
| - Rectum Adenocarcinoma | -1.586 | 5.1E-05 |

**Supplementary Table S2: Primer sequences used for real time qPCR analysis**

| Gene | Specie | Primer forward (5′ - 3′) | Primer reverse (5′ - 3′) |
|---|---|---|---|
| *Ripk3* | human | TTTGGCCTGTCCACATTTCAG | GGTTGGCAACTCAACTTCTCTT |
| *Ll32* | human | TGTCCTGAATGTGGTCACCTGA | CTGCAGTCTCCTTGCACACCT |
| *Ll32* | mouse | GAAACTGGCGGAAACCCA | GGATCTGGCCCTTGAACCTT |
| *Tnfa* | mouse | CATCTTCTCAAAATTCGAGTGACAA | TGGGAGTAGACAAGGTACAACCC |
| *Il6* | mouse | CTGCAAGAGACTTCCATCCAGTT | GAAGTAGGGAAGGCCGTGG |
| *Il1β* | mouse | CGGCACACCCACCCTG | AAACCGCTTTTCCATCTTCTTCT |
| *Il11* | mouse | CTGCACAGATGAGAGACAAATTCC | GAAGCTGCAAAGATCCCAATG |
| *Ifng* | mouse | AGCTCATCCGAGTGGTCCAC | GCTTCCTGAGGCTGGATTCC |
| *Ccl2* | mouse | GCTGGAGCATCCACGTGTT | ATCTTGCTGGTGAATGAGTAGCA |
| *Cxcl1* | mouse | AATGAGCTGCGCTGTCAGTG | TGAGGGCAACACCTTCAAGC |
| *Cxcl2* | mouse | CCTGCCAAGGGTTGACTTCA | TTCTGTCTGGGCGCAGTG |
| *S100a9* | mouse | GGTGGAAGCACAGTTGGCA | GTGTCCAGGTCCTCCATGATG |
| *Ereg* | mouse | CACCGAGAAAGAAGGATGGA | GATTCTCCTGGGATGCATGA |
| *Mmp10* | mouse | CACAAGCCCAGCTAACTTCC | TTTGTCTGGGGTCTCAGGTC |
| *Cox2* | mouse | TGAGCACAGGATTTGACCAG | CCTTGAAGTGGGTCAGGATG |
| *Hif1a* | mouse | TGCTCATCAGTTGCCACTTC | CCATCTGTGCCTTCATCTCA |
| *Ido* | mouse | CTGCCTGTGCTGATTGAGAA | CCTTTCGAACATCGTCATCC |
| *Wnt5a* | mouse | GGTGCCATGTCTTCCAAGTT | TGAGAAAGTCCTGCCAGTTG |
| *Wisp1* | mouse | CAGATGGCTGTGAATGCTGT | AAGGACTCGCCATTGGTGTA |
| *cMyc* | mouse | TCTCCACTCACCAGCACAACTACG | ATCTGCTTCAGGACCCT |
| *Cyclin D1* | mouse | CCCTGACACCAATCTCCTCAAC | GCATGGATGGCACAATCTCCT |
| *Cyclin E* | mouse | ATGTGGCCGTGTTTTGCA | GGTCTGATTTTCCGAGGCTGA |
| *Cyclin B1* | mouse | ACTTCAGCCTGGGTCGCC | ACGTCAACCTCTCCGACTTTAGA |
| *Bclxl* | mouse | GGTCGCATCGTGGCCTTT | TCCGACTCACCAATACCTGCAT |
| *p21* | mouse | ATTCAGAGCCACAGGCACCAT | TCTCCGTGACGAAGTCAAAGTT |
